# Eccentric Exercise: Adaptations and Applications for Health and Performance

**DOI:** 10.3390/jfmk6040096

**Published:** 2021-11-24

**Authors:** Michael O. Harris-Love, Jared M. Gollie, Justin W. L. Keogh

**Affiliations:** 1 Physical Therapy Program, Department of Physical Medicine and Rehabilitation, University of Colorado Anschutz Medical Campus, Aurora, CO 80045, USA; 2 Geriatric Research Education and Clinical Center, VA Eastern Colorado Healthcare System, Aurora, CO 80045, USA; 3 Muscle Morphology, Mechanics, and Performance Laboratory, Geriatrics Service, Veterans Affairs Medical Center, Washington, DC 20422, USA; Jared.Gollie@va.gov; 4 Department of Health, Human Function, and Rehabilitation Sciences, School of Medicine & Health Sciences, George Washington University, Washington, DC 20052, USA; 5 Faculty of Health Sciences and Medicine, Bond University, Robina, QLD 4226, Australia; jkeogh@bond.edu.au; 6 Sports Performance Research Centre New Zealand, Auckland University of Technology, Auckland 1010, New Zealand; 7 Cluster for Health Improvement, Faculty of Science, Health, Education and Engineering, University of the Sunshine Coast, Sunshine Coast, QLD 4556, Australia; 8 Kasturba Medical College, Mangalore, Manipal Academy of Higher Education, Manipal 576104, Karnataka, India

**Keywords:** eccentric training, resistance training, rehabilitation, strength, power, hypertrophy, tendinopathy, youth athletes, aging, flywheel training

## Abstract

The goals of this narrative review are to provide a brief overview of the muscle and tendon adaptations to eccentric resistance exercise and address the applications of this form of training to aid rehabilitative interventions and enhance sports performance. This work is centered on the author contributions to the Special Issue entitled “Eccentric Exercise: Adaptations and Applications for Health and Performance”. The major themes from the contributing authors include the need to place greater attention on eccentric exercise mode selection based on training goals and individual fitness level, optimal approaches to implementing eccentric resistance exercise for therapeutic purposes, factors that affect the use of eccentric exercise across the lifespan, and general recommendations to integrate eccentric exercise in athletic training regimens. The authors propose that movement velocity and the absorption or recovery of kinetic energy are critical components of eccentric exercise programming. Regarding the therapeutic use of eccentric resistance training, patient-level factors regarding condition severity, fitness level, and stage of rehabilitation should govern the plan of care. In athletic populations, use of eccentric exercise may improve movement competency and promote improved safety and performance of sport-specific tasks. Eccentric resistance training is a viable option for youth, young adults, and older adults when the exercise prescription appropriately addresses program goals, exercise tolerability, and compliance. Despite the benefits of eccentric exercise, several key questions remain unanswered regarding its application underscoring the need for further investigation.

## 1. Introduction

Eccentric muscle actions—which yield net force production during active muscle lengthening—remain both a scientific curiosity and a ubiquitous element of mobility and task performance. Over 90 years have passed since A. V. Hill and his protégé, Wallace Fenn, provided keen insights into what physiologists now regard as the “negative Fenn effect”, whereby a given force produced through eccentric muscle actions requires lower metabolic cost in comparison to isometric and concentric muscle actions [1]. Incremental advances have given rise to important findings regarding the bioenergetics of eccentric muscle actions and the peculiarities of the force–velocity curve during “negative work” exercises [2,3]. Methods ranging from molecular approaches and various bioimaging techniques to mechanical modeling have led to important lines of investigation, including the “winding filament” hypothesis and the role of titin in active force enhancement, the impact of in vivo muscle mechanics on eccentric force production, variation in neuromuscular activation strategies based on muscle action mode, and the differential morphological muscle and tendon adaptions that result from chronic eccentric muscle actions [3,4,5,6,7].

Importantly, the unique characteristics of eccentric muscle actions have stimulated interest in the use of eccentric exercise in a wide variety of experimental and applied settings. The application of eccentric exercise has evolved from a model to induce muscle damage under laboratory conditions, to selected forms of strengthening exercise used to enhance sports performance, and most recently as an approach to identify musculoskeletal injury risk in sport and as a form of therapeutic exercise for clinical and athletic populations [8,9,10]. Despite advancements in the understanding of the benefits of eccentric exercise, fundamental questions regarding appropriate implementation and optimal exercise prescription remain. This Special Issue of the *Journal of Functional Morphology and Kinesiology* addresses some of the evidence gaps concerning the mechanisms of muscle and tendon adaptations to eccentric exercise and the emerging applications of this unique form of exercise. In this introduction to the Special Issue, the editors present a narrative review highlighting the major themes from the contributing authors. These collective works make important distinctions across multiple modes of eccentric exercise, examine the adaptations to active muscle lengthening for therapeutic purposes, consider the use of eccentric exercise across the lifespan, and provide recommendations to integrate eccentric exercise in athletic training regimens.

## 2. Eccentric Exercise: One Muscle Action, Two Uses of Kinetic Energy, Many Modes of Exercise

The net force generated during active muscle lengthening occurs when the external resistance exceeds momentary force produced by the agonist muscle [1,11]. Eccentric muscle actions are governed by the complex integration of viscoelastic behavior of the muscle-tendon unit (MTU) and residual force enhancement. Residual force enhancement associated with active muscle lengthening is not completely explained by cross-bridge interactions or passive MTU elements [3,4], and may be augmented by Ca^++^ mediated changes in the stiffness of sarcomeric proteins such as titin [4,12,13].

**Figure 1:**
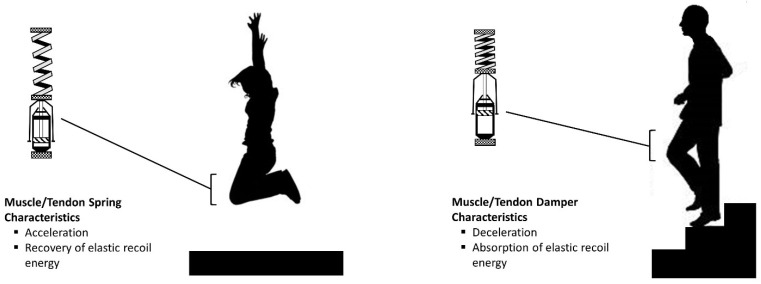
Muscle and tendon mechanics during eccentric muscle actions reflect a damper in series with a spring. The rapid stretch of muscles and tendons in the loading phase of a vertical jump yields stored elastic recoil energy that is released during the flight phase of the movement (i.e., extension of the spring). In contrast, descending stairs at customary speed typically results in the deceleration of joint motion during the controlled lowering phase of stair negotiation with the absorption of elastic recoil energy lost as heat (i.e., absorption via the damper). These muscle and tendon characteristics are influenced by movement velocity, force magnitude, and tissue stiffness. Figure adapted from Lindstedt et al. [1].

Muscle performance during activities that emphasize active muscle lengthening is influenced by angular joint velocity, instantaneous muscle stiffness, as well as the timing and magnitude of the external resistance or imposed force [1]. Stan Lindstedt notably provided the example of the “shock absorber” (i.e., damper) in series with a spring to characterize the performance spectrum of eccentric muscle actions [1,14]. Eccentric exercise should be conceptualized as two distinct categories of activity with differing uses of kinetic energy (Figure 1). Based on the *Lindstedt model* of a spring in series with a damper, these eccentric exercise categories are largely defined by the function of kinetic energy [1,10,15]:

(1) *Recovery of Kinetic Energy:* Activities that potentiate force production via ballistic movements involving maximal acceleration with very short surface contact time. These repetitive activities facilitate the recovery of elastic recoil energy and contribute to the coupling phases of the stretch-shortening cycle (SSC) [15,16].
(2) *Absorption of Kinetic Energy:* Activities that typically result in the deceleration of angular joint velocity during non-ballistic movements. These activities result in eccentric force production and the absorption of kinetic energy which is dissipated as heat [4,17].

**Figure 2:**
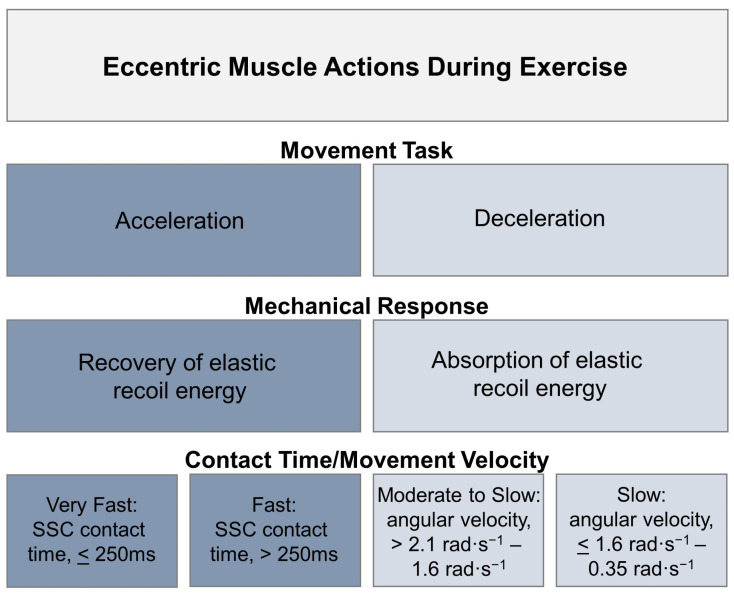
Exercise methods that emphasize eccentric muscle actions may be categorized by the general movement task and the function of kinetic energy. Eccentric exercise activities defined by either the storage and recovery of kinetic energy (e.g., elastic recoil energy) or the absorption of kinetic energy may constitute distinct forms of eccentric exercise with differing physiological adaptations. Figure adapted from Harris-Love et al. [10] and Vogt and Hoppeler [15] (SSC, stretch-shortening cycle; rad, radians).

Consequently, it is critical that the general interpretation of the findings of literature reviews, commentaries, and meta-analysis studies consider these distinct categories of eccentric exercise and not just parameters such as workload, frequency, or volume (Figure 2). Conventional programming parameters remain essential to characterizing eccentric exercise regimens [10]. However, the temporal features of muscle activation during eccentric muscle actions and the movement velocity of the given exercise activity also drive the demands placed on the MTU and influence the expected physiologic adaptations in response to eccentric exercise [9,10,15,18]. Previous commentators have recognized that eccentric exercises that involve decelerating external loads are ideal for the development of maximal and explosive strength capacity in athletes such as alpine skiers [15]. Eccentric exercise involving the deceleration of external loads has also been used for the rehabilitation of people with chronic musculoskeletal conditions [10]. In contrast, exercises that involve maximal acceleration and rapid eccentric muscle activation, such as plyometric exercises, involve adaptions of the SSC which are favorable for athletic events involving sprinting, jumping, and other explosive maneuvers [16].

The recent reviews by Suchomel et al. [19,20] are instructive regarding the consideration of eccentric exercise modes based on the physical performance goals, individual training status, and demands and constraints of the exercise. The authors highlight that the absorption of kinetic energy, and the resultant overload stimulus applied to the MTU, differ with varied modes of eccentric exercise which include: tempo eccentric training, flywheel overload training, accentuated eccentric loading, and plyometric training [20]. Consequently, exercise-induced adaptations in muscle hypertrophy, peak force production, and power vary with differing eccentric training methods (Table 1).

Plyometric training is well known to induce physiologic adaptations that emphasize peak muscle power development [16,19], whereas flywheel overload training and tempo eccentric training may be relatively advantageous for increasing muscle hypertrophy [19,21]. Suchomel et al. and other commentators [19,20,22] correctly note that sport activities typically incorporate a combination of both concentric and eccentric muscle actions, and that some modes of exercise with a significant component of active muscle lengthening may be applied along a continuum of kinetic energy storage and absorption [19]. Accentuated eccentric loading features coupled concentric and eccentric muscle action phases with increased loading during active muscle lengthening [19,23]. The undisrupted concentric phase coupled with the eccentric overloading phase allow for both high magnitudes of mechanical tension and potential increases in the eccentric rate of force development [19,20]. Additional studies are needed to better understand the neuromuscular and functional advantages of accentuated eccentric loading in comparison to traditional progressive resistance exercise. Moreover, further evidence is needed to ascertain the optimal eccentric stimulus to employ relative to individual strength levels. Nevertheless, the early evidence suggests that accentuated eccentric loading is a versatile mode of exercise that may have value in developing muscle hypertrophy, peak force, and power [19].

## 3. Eccentric Exercise as a Therapeutic Intervention

The use of eccentric exercise as a therapeutic intervention has gained increased acceptance over time [3,4,10,11], with early applications employed to treat tendinopathies [7] and gradually expanding to feasibility and efficacy studies involving a variety of chronic conditions [9,24]. This transition has been marked by applied use of muscle action history to mitigate potential adverse effects of eccentric overload and the phased approach to submaximal loading in clinical populations and untrained individuals [9,10]. A phased approach to eccentric exercise that includes familiarization, acclimatization, and progression phases has been proposed for people with chronic conditions [10,25]. Low initial workloads and movement velocities allow untrained people who are either naïve to eccentric overload stimuli or limited by chronic conditions to significantly decrease in motor performance variability during a familiarization phase that includes 2 to 3 exercise bouts [10]. Moreover, use of a 1 to 2 week acclimatization phase has been shown to allow participants with significant knee osteoarthritis to engage in progressively higher isokinetic knee extension/flexion workloads without adverse events secondary to the intervention [10,26]. Similarly, the incorporation of a phased approached in older adults with moderate-to-severe chronic kidney disease allowed for the identification and progression of power output when using flywheel resistance exercise to elicit eccentric overload [27]. While athletes and other physically active individuals may be able to quickly acclimate to a novel exercise task that emphasizes active muscle lengthening, the injury risk profile and time course for exercise-induced MTU adaptations differ for clinical populations [25,28]. Indeed, muscle action history characterized by high force production results in greater exercise induced muscle damage and longer periods of the protective repeated bout effect [29,30,31]. However, people with chronic conditions may require relatively lower forces over a longer span of time to induce an adequate repeated bout effect sufficient to engage in higher intensity eccentric exercise. Seminal work regarding the use of eccentric exercise as a form of therapeutic intervention include a study conducted by Meyer et al. [32] concerning the effective implementation of an eccentric cycling regimen for people with coronary artery disease (CAD). Study participants with CAD in the eccentric cycling group completed over 300% more total work in comparison to participants who were in the concentric cycling group. Despite the magnitude of this difference, the two groups exhibited similar hemodynamic responses to exercise [32]. Safe use of eccentric exercise has been demonstrated for people with a wide range of conditions including Parkinson disease, arthritic conditions, pulmonary disease, and inflammatory muscle disease [9,24,25]. Investigators have reported increased mobility in a sample of community-dwelling older adults upon completion of an eccentric ergometry program [33], and post-regimen improvements in walking speed have been noted in individuals with knee osteoarthritis (OA) engaged in combined concentric-eccentric strengthening exercise [34]. However, a recent investigation comparing accentuated eccentric strengthening exercise with standard strengthening exercise for people with knee OA suggested that there were no differences in functional outcomes between the two exercise groups [35].

While the efficacy of eccentric muscle actions used as therapeutic interventions is often viewed through the lens of muscle adaptations, early applied work within this field involved the treatment of tendinopathies [7,36]. The mechanism of tendon tissue remodeling secondary to eccentric exercise includes the response of tenocytes to strain which results in adaptations such as increased collagen synthesis and normalized collagen morphology [36,37]. These adaptations may be facilitated via the post-exercise upregulation of transforming growth factor-β-1 (TGF-β-1), insulin growth factor-1Ea (IGF-1Ea), and mechano growth factor (MGF), as well as collagen type 1 and type 3 [37]. Elements of the eccentric exercise prescription such as slower movement velocities and relatively high workloads, may affect the magnitude of post-exercise tendon tissue adaptations. The work by Quinlan et al. [37] highlights that tendon tissue adaptions to an eccentric exercise stimulus are proportional to magnitude of workload and strain. Moreover, it has been observed that exercise regimens involving relatively low workloads are comparatively less effective at inducing tendon tissue adaptations than routines that incorporate higher workloads [37,38]. Nevertheless, questions remain concerning if the nature of eccentric muscle actions confer any benefit over concentric muscle actions, apart from workload magnitude, when employing therapeutic exercise to treat tendinopathies. The review presented by Jayaseelan et al. [36] mirrors this point as their findings suggested that eccentric exercise interventions for tendinopathies demonstrate efficacy in comparison to non-intervention control groups, but conclusions are equivocal when comparing eccentric exercise to other forms of exercise or multimodal interventions. Notably, Jayaseelan and colleagues [36] highlight the Load-Induced Tendinopathy Continuum Model as a structured approach to determine the ideal exercise mode based on the severity of the condition and other patient factors [39]. The authors recommend that earlier phases of the recovery continuum may merit a more conservative approach to therapeutic exercise whereas late phase rehabilitation geared towards the return to relatively demanding physical activities (e.g., running and jumping) would benefit from the integration of eccentric exercise into the management of tendinopathy [36]. Future investigations concerning the use of eccentric exercise for a variety of conditions should further explore patient-centered factors and phases of recovery to drive the selection of exercise mode across the continuum of care.

## 4. Eccentric Exercise across the Lifespan

Resistance exercise offers numerous health and functional benefits in both young and aging populations [40,41,42]. Despite the known benefits of resistance exercise, there is much to be understood regarding the implementation of eccentric exercise in these individuals. For example, eccentric exercise is perceived as being important for youth athletes even though empirical evidence supporting its application is currently limited [43]. Injury prevention is the primary rationale for the inclusion of eccentric exercises in youth athletes followed by change of direction, strength and power, injury rehabilitation and muscle hypertrophy [43]. Based on a survey conducted in sport coaches, the decision to include eccentric exercise into a training regime for youth athletes was most influenced by (1) movement competency, (2) training age, and (3) maturation status [43]. The magnitude of exercise induced muscle damage in response to eccentric resistance exercise is less in youth in comparison to younger adults in both males and females [44,45,46]. However, further research is required to uncover the factors contributing to differences in eccentric exercise-induced muscle damage across various age ranges. In addition, the repeated bout effect is present in younger individuals but does not seem to be influenced by maturation age [45].

It is well established that force generating capacity declines with age and contributes to reductions in functional capabilities [47,48,49,50]. Interestingly, age-associated declines in eccentric force are less than those observed for concentric force [51]. The finding of slower decline in eccentric force with aging, in combination with the unique neuromuscular responses to muscle lengthening overload described above, has made eccentric exercise an appealing treatment option for maintaining and/or improving neuromuscular health and physical function in older adults [21,37,52]. Similar to findings in youth, older adults experience less exercise induced muscle damage in response to eccentric overload when compared to younger adults [30,53]. Conversely, while the repeated bout effect is found to be present in older adults, the magnitude of the response is less than that observed in younger adults [30,53]. The effects of eccentric exercise in older adults has demonstrated improvements in skeletal muscle structure, strength, power, balance, stair descent, and fall risk [33,54,55,56,57].

The reviews by Kowalchuk and Butcher [21] and Quinlan et al. [37] highlight greater force production and low energetic cost of muscle lengthening contractions as justification for the application of eccentric exercise in older adults. Kowalchuk and Butcher [21] describe the potential benefits of eccentric resistance exercise using flywheel technology in older adults. They specifically note preliminary evidence that the use of eccentric overload resistance exercise using flywheel devices by older adults may result in improved muscle power and postural stability [21,58]. While the initial findings using flywheel resistance exercise in older adults seem promising, the authors acknowledge the need for further investigation due to the paucity of evidence currently available. In addition, Quinlan et al. [37] detailed the potential implications of eccentric exercise in older adults on tendon-specific adaptations. It is not clear if eccentric resistance training offers a superior stimulus for tendon adaptations compared to traditional resistance training. The authors hypothesize that eccentric exercise could accelerate the time course of tendon adaptations due to the greater strain imposed. However, it is important to consider both the potential risks and benefits of eccentric exercise when working with the aging population. For example, subjecting older adults to high eccentric loads may increase the potential for injury when applied to an already compromised MTU. Such concerns may be mitigated with the inclusion of an initial acclimatization period using lighter loads and progressing to higher eccentric loads as the MTU adapts [9,10,59]. Overall, eccentric exercise seems to provide a safe treatment option for older adults to enhance force generation and physical functioning. Strong considerations should be given to the exercise modality, intensity, and rate of progression when prescribing eccentric exercise in older adults to maximize tolerability and compliance.

## 5. Integrating Eccentric Exercise into Sports Training

While there is now considerable evidence regarding the importance of high levels of eccentric strength for sporting activities requiring acceleration and deceleration such as jumping, sprinting and change of direction tasks (e.g., cutting as well as reducing the risk of injury), the question of how eccentric exercise should be integrated into overall sports training is still less well understood. In particular, some of the primary questions practitioners may continually grapple with include: (1) how to limit the degree of delayed onset muscle soreness and fatigue post eccentric training, (2) what type of eccentric training should be used with their athletes across different phases of the periodization plan, and (3) how a variety of athlete characteristics such as age, sex, sport and training history may influence what constitutes their optimal form of eccentric training. Two recent papers have reviewed the relevant literature in an attempt to provide an overview of the potential benefits of eccentric training for team sport [60] and youth athletes [46] and to provide some practical recommendations regarding the integration of eccentric training into the overall training programs for these athletic groups.

McNeil and colleagues [60] conducted a systematic review involving 14 studies of team sport athletes from basketball, soccer, handball or rugby union, who performed a minimum of three weeks of either eccentric overload or accentuated eccentric resistance training. These forms of eccentric resistance training typically produced moderate (effect size = 0.6–1.2) increases in muscular strength, muscular power, sprinting speed and change of direction ability, with the percent change typically greater for muscular strength and change of direction ability compared to muscular power and sprinting speed [60]. However, it should also be acknowledged that a number of these studies also found trivial to small changes in these outcome measures [60]. The relative equivalence of some of these results suggest that future research should explore how the adaptations to eccentric training may be influenced by the team sport athletes’ baseline eccentric capabilities. Additional research should also address how eccentric training adaptations are impacted by specific training variables (some of which were not adequately described in the reviewed studies) to better characterize the dose response to eccentric training. Nevertheless, McNeil and colleagues [60] recommend that eccentric resistance training should be included as a component of the overall resistance training programs of team sport athletes, with a number of caveats. These include:

(1) While inertial flywheel training can be an effective form of eccentric overload training, the magnitude of the eccentric peak force is influenced by the effort and intention of the trainee, with trainees with more experience in flywheel training producing greater peak eccentric force [61]. This may mean that only resistance trained team sport athletes with experience in flywheel training will be able to produce sufficient eccentric overload and therefore benefit from using this form of training. However, it may also mean that such training could be a useful way to introduce lower intensity eccentric training to younger athletes or those with less resistance training experience.
(2) There is the possibility of a velocity specificity of eccentric training (Figure 2), in which high-speed eccentric training may provide a better training stimulus for activities involving fast eccentric actions such as sprinting and jumping than low-speed eccentric training. However, even if this velocity specificity exists, it may still be useful to prescribe a variety of eccentric exercises involving slower and faster muscular actions to improve team sport athletes’ movement capacities.

Drury et al. [46] performed a narrative review to determine the potential application of eccentric resistance training for youth athletes and how this might best be utilized as a component of their long-term athletic development. This review summarized a number of the neuromuscular and metabolic responses to eccentric exercise in youth, including the repeated bout effect that may be important factors to consider when looking to incorporate eccentric training for this population [46]. Due to the potential for fatigue, muscle damage or injury with high load eccentric loading, the review also examined how eccentric resistance training may influence fatigue and muscle damage in youth athletes [46]. Based on the 12 identified studies that assessed the fatigue and muscle damage associated with eccentric training, it appeared that youth athletes experienced less exercise-induced muscle damage following eccentric exercise than adults [46]. (See Section 4 for additional commentary on lifespan factors associated with eccentric training.) On the basis of this relative safety and the demonstrated benefits of eccentric training for youth athletes [62,63], Drury et al. [46] have made the following recommendations for how different forms of eccentric resistance training could best be included in the long-term athlete development training plans of youth athletes:

(1) Consistent practicing of progressively more challenging hopping and jumping tasks by athletes, in which landing mechanics are emphasized. This focus on landing mechanics is important so athletes develop the ability to absorb eccentric forces and improve their movement competency. This approach facilitates improved safety and performance of complex motor skills involved in sport-specific tasks.
(2) Eccentric hamstring strength development should be emphasized to improve youth athletes running and jumping performance and reduce the risk of injury while performing these motor tasks. Such training could involve the Nordic hamstring curl as well as more hip extensor dominant hamstring exercises such as 45° hip extensions. All of these eccentric hamstring exercises can significantly increase eccentric strength and muscle fascicle lengths [64], with such adaptations related to substantial reductions in the risk of hamstring injury [65].
(3) Inertial flywheel training is another option to increase eccentric strength for youth athletes, with some studies demonstrating such exercises produced significant improvements in youth athletes’ jumping, sprinting and change of direction ability. However, low intensity flywheel exercise should be performed initially using flywheels with lower inertia wheels than that used with adult athletes in the same sport.

It was also apparent that Drury et al. [46] were aware that such training recommendations need to be matched to the athlete’s level of development and training history. Thus, Drury et al. [46] provided additional practical recommendations on how these forms of eccentric exercise might best be incorporated and progressed over the youth athletes’ maturation from pre-, during- and post-peak height velocity. If such progressions in exercise selection, load, sets, repetitions, movement velocity are followed, then it appears that these forms of eccentric training may be safely incorporated across the youth athletes’ developmental journey and result in improved performance outcomes.

## 6. Conclusions

The purpose of this narrative review is to provide a brief overview of the muscle and tendon adaptations to eccentric resistance exercise and consider the applications of this form of training in rehabilitation and athletic performance settings. The key papers featured in this work are from contributors to the *Journal of Functional Morphology and Kinesiology* Special Issue entitled “Eccentric Exercise: Adaptations and Applications for Health and Performance”. This Special Issue features a total of 8 papers, encompassing 28 different affiliations, with authors from 7 different countries spanning Europe, Australia, and North America.

Recent special journal issues and systematic reviews concerning eccentric exercise have focused on the mechanisms associated with tissue adaptations to chronic active muscle lengthening stimuli, spinal and supraspinal control of eccentric muscle actions, biomarkers of post-eccentric muscle action damage, use of eccentric exercise training for rehabilitative purposes, and the low energy expenditure relative to force output during eccentric muscle actions [3,15,22,24,66]. These recent works uniformly accept the premise that “the early doctrine espousing eccentrics as dangerous and having no clinical usefulness has now been replaced” [4]. Indeed, a phased approach to integrating eccentric exercise into therapeutic exercise regimens or sports-specific training plans that involves managing workload, movement velocity, and the repeated bout effect are broadly recognized [4,9,10,11,15,33,46,59]. However, a view shared in recent reviews is that common recommendations or guidelines are lacking for eccentric exercise [4,10,22]. This critique has to be measured against the steep challenge of generating specific eccentric exercise recommendations across a wide variety of pathological conditions or sport-specific needs. Rather, the use of eccentric exercise for patient populations has to be driven by patient-level frameworks such as the Load-Induced Tendinopathy Continuum Model as conveyed by Jayaseelan et al. [36] and rehabilitation-specific approaches to eccentric exercise familiarization and inducement of the repeated bout effect as proposed by the authors and other investigators [10,59]. 

Rational direction for the wider use of eccentric exercise with athletic populations may be taken from the works of Drury et al. [46] and McNeil et al. [60]. Emphasizing eccentric muscle actions used in the functional application of progressive hopping and jumping tasks, along with eccentric resistance training using movement velocities consistent with sport-specific tasks, are sound exercise programming principles to employ in a variety of sports settings [46]. The modality of eccentric exercise being applied further complicates the challenge of establishing widely accepted prescription guidelines across various populations. The two part review by Suchomel et al. [19,20] highlights methodological differences and practical implications of tempo eccentric training, flywheel inertial training, accentuated eccentric loading, and plyometric training. Similarly, reviews by Kowalchuk and Butcher [21] and Quinlan et al. [37] extend the discussion of eccentric exercise in older adults to include the application of flywheel inertial training and considerations for tendon adaptations. Collectively, the *Journal of Functional Morphology and Kinesiology* Special Issue entitled “Eccentric Exercise: Adaptations and Applications for Health and Performance” adds to a growing literature supporting eccentric exercise as a viable option across the lifespan and in sport performance for enhancing force characteristics, injury prevention, and improved functional capacity. Importantly, despite the positive findings outlined in this Special Issue, several key questions remain unanswered regarding the application of eccentric exercise underscoring the need for further investigation.

## Figures and Tables

**Table 1:** Summary of underlying eccentric training effects that may benefit hypertrophy, strength, and power output. Arrows represent an increase (or decrease) in the specific outcome of interest in response to eccentric training.

Hypertrophy	Strength	Power Output
↑Anabolic signaling ↑Satellite cell activation ↑Motor unit recruitment ↑Activation of motor cortex ↑Force production capacity Possible ↑ fast twitch motor unit preferential recruitment	↑Activation of motor cortex ↑Force production ↑Motor unit discharge rate ↑Muscle-tendon unit stiffness ↓Regulation of inhibitory reflexes Possible ↑fast twitch motor unit preferential recruitment Possible ↑type IIx fiber composition (phenotype shift)	↑Motor unit recruitment ↑Activation of motor cortex ↑Force production capacity ↑Motor unit discharge rate ↑Muscle-tendon unit stiffness ↓Regulation of inhibitory reflexes ↑Muscle fascicle length Possible ↑ fast twitch motor unit preferential recruitment Possible ↑ type IIx fiber composition (phenotype shift) Possible ↑ excitation-contraction couple rates ↑Muscle fiber shortening velocity

Reproduced with permission from Suchomel et al. [19] *J. Funct. Morphol. Kinesiol.* 2019, *4*(2), 38; https://doi.org/10.3390/jfmk4020038.

## Data Availability

Not applicable.
